# Characterization and Comparison of Biodegradable Printed Capacitive Humidity Sensors

**DOI:** 10.3390/s21196557

**Published:** 2021-09-30

**Authors:** Emma Wawrzynek, Carol Baumbauer, Ana Claudia Arias

**Affiliations:** Department of Electrical Engineering and Computer Science, University of California, Berkeley, CA 94720, USA; efwawrzynek@berkeley.edu (E.W.); carol_baumbauer@berkeley.edu (C.B.)

**Keywords:** humidity sensor, biodegradable sensor, capacitive sensor, inkjet printed, interdigitated electrodes (IDE), relative humidity

## Abstract

Flexible and biodegradable sensors are advantageous for their versatility in a range of areas from smart packaging to agriculture. In this work, we characterize and compare the performance of interdigitated electrode (IDE) humidity sensors printed on different biodegradable substrates. In these IDE capacitive devices, the substrate acts as the sensing layer. The dielectric constant of the substrate increases as the material absorbs water from the atmosphere. Consequently, the capacitance across the electrodes is a function of environmental relative humidity. Here, the performance of polylactide (PLA), glossy paper, and potato starch as a sensing layer is compared to that of nonbiodegradable polyethylene terephthalate (PET). The capacitance across inkjet-printed silver electrodes is measured in environmental conditions ranging from 15 to 90% relative humidity. The sensitivity, response time, hysteresis, and temperature dependency are compared for the sensors. The relationship between humidity and capacitance across the sensors can be modeled by exponential growth with an R^2^ value of 0.99, with paper and starch sensors having the highest overall sensitivity. The PET and PLA sensors have response and recovery times under 5 min and limited hysteresis. However, the paper and starch sensors have response and recovery times closer to 20 min, with significant hysteresis around 100%. The PET and starch sensors are temperature independent, while the PLA and paper sensors display thermal drift that increases with temperature.

## 1. Introduction

Humidity sensing is critical to a wide range of applications, from manufacturing to healthcare. The need for cheap and versatile humidity sensors is extensive [1] and can be met by recent innovations in printed flexible electronics [2]. The adaptability of printing methods allows for sensors to be made with unique substrates, such as paper [3,4], egg whites [5], and onion skins [6]. These types of biodegradable humidity sensors are of particular interest for applications in smart packaging and agriculture.

Smart packaging utilizes sensors fabricated directly into packaging material in order to track information about its contents and environmental history, such as pH, temperature, gas levels, light exposure, and relative humidity [7,8,9]. Schaefer and Cheung [10] identify key challenges in the development of smart packaging, including fabricating thin film technology and reducing the environmental impact of unsustainable packaging materials. This work addresses these challenges by presenting printed humidity sensors that function on a range of biodegradable packaging material.

Another use for biodegradable humidity sensors is in high density spatial and temporal monitoring of growing conditions. Extensive data on a crop’s condition allow for more efficient use of resources, and the use of biodegradable sensors can reduce the impact of electrical and plastic waste, as well as the labor required to retrieve sensors at the end of a crop cycle [11,12].

Currently, it is challenging to compare the performance of humidity sensors printed on different substrates because of a lack of standard sensor characterization and fabrication. Not only are sensors made in different ways [6,13], but they are also often tested under different conditions [3,4,14]. In this paper, we directly compare the performance of a range of printed sensors that are fabricated and tested via a standard method.

Humidity can be quantified in many ways, two of which are absolute and relative humidity (RH). Relative humidity is the most common measurement for environmental humidity and is the ratio of actual water vapor partial pressure to the saturation vapor pressure. The saturation vapor pressure is the point at which condensation occurs. Saturation vapor pressure is an exponential function of temperature, in that hotter air can hold more water vapor. The mass of water vapor per volume of air is called absolute humidity and, unlike relative humidity, it is not dependent on temperature [15]. This means that the same absolute humidity can be achieved by various combinations of relative humidity and temperature. For example, at standard pressure, 15 °C air at 90% relative humidity contains the same amount of water vapor per volume as 25 °C air at 50% relative humidity, thus both conditions share the same absolute humidity.

Printed humidity sensors typically take the form of interdigitated electrodes (IDEs), with capacitance measured between the two interwoven electrodes. The capacitance of an IDE depends on the number of fingers, finger width, gap width, and finger overlap, as well as the dielectric constant and thickness of the substrate on which it is printed. An IDE sensor indirectly measures humidity as a function of water vapor absorbed by the sensor’s substrate. In humid environments, the substrate absorbs water, which has a relatively high dielectric constant compared with that of the substrate. As a result, the dielectric constant of the material increases, causing a proportional increase in the capacitance reading across the sensor. When relative humidity drops, water vapor evaporates out of the substrate to achieve equilibrium, and the capacitance decreases.

While IDE capacitance varies linearly as a function of substrate dielectric constant [16], the absorption and desorption of water vapor into a substrate is nonlinear and highly dependent on the substrate properties. As a result, capacitive output is not linearly related to relative humidity [17]. The sensor’s capacitive response relies on the permeation of environmental water vapor through the substrate. Water molecules must absorb to the sensor’s surface, diffuse through the substrate, and finally desorb when humidity drops. The permeation process is altered by properties such as the material polarity, thickness, porosity, and the presence of an external coating. If a material is made up of polar chemical groups, it will form strong interactions with polar water molecules, which leads to changes in absorption behavior. For a polar substrate, the rate of diffusion of water vapor increases with an increase in absorbed molecules. This is because the polar–polar interactions between substrate and water cause swelling, which in turn introduces more free volume into the material. Free volume provides space for absorbed molecules to reside, as well as paths for molecules to move through the substrate [18,19,20,21,22].

Substrate choice is fundamental to the behavior of IDE capacitive humidity sensors. This paper characterizes and directly compares sensors printed on four different materials: planarized polyethylene terephthalate (PET), polylactic acid (PLA), glossy photo paper, and a starch and polypropylene (PP) composite.

PET is a polar polymer that is commonly used for packaging of foods and liquids. It is also a standard material that can be optimized for printed, flexible electronics, and has been studied as a base substrate for humidity sensors [23,24]. Unlike the other three substrates, it is not biodegradable. However, PET is a popular synthetic polymer that is slowly being replaced by environmentally friendly alternatives such as PLA, and thus is included as a control material in this study.

PLA is also a polar polymer, but is made from plant matter and is biodegradable. It was chosen for this study because it is highly ubiquitous, and often considered a biodegradable alternative to PET. PLA is a popular material for packaging and degradable medical devices and is one of the most common filaments for 3D printing. PLA can be degraded by hydrolysis, photo, microbial, and enzymatic degradation. Under ambient conditions in air and soil, it degrades slowly [25,26,27].

Paper is another popular material for humidity sensors as it is versatile and affordable. A potential application for printed humidity sensors is monitoring shipments of food or medicine, in which sensors can be directly printed onto paper labels or cardboard boxes. Furthermore, many sensors for environmental monitoring are already paper-based, allowing for the potential development of an array of environmental sensors printed on a single sheet of paper. Humidity sensors could be integrated with already established air quality monitoring and electrochemical detecting paper sensors [28]. Paper can be broken down in a variety of ways, and recycling infrastructure already exists to process the large amounts of paper consumed by humans. Besides recycling, paper can be chemically degraded by acid-catalyzed hydrolysis and oxidation or composted and broken down by bacteria in soil. In order to prevent water from quickly degrading paper, the substrate needs to be protected from environmentally introduced acids and made from the appropriate raw materials. Thus, if used under the right conditions, paper-based humidity sensors have the potential to last for years [29]. In this study, photopaper is used because its glossy coating prevents ink from spreading and allows for high resolution inkjet printed features. Typically, photopaper is acid-free in order to extend its lifespan.

Finally, the starch and PP composite is a polar, commercially available bioplastic sold in the form of “Tater Ware”. It is comprised primarily of potato starch and used to make cutlery and other tableware. Starch can be combined with a variety of petroleum-based or biopolymers to make inexpensive, unique polymer combinations. For this reason, starch has recently become popular for use in developing new sustainable plastics [30], and thus is an important material to study. All starch-based bioplastics are degradable via hydrolysis as well as microbial activity. Changing the porosity of the composite can consequently affect the lifespan, as highly porous materials are more prone to microbial degradation.

In this paper, the substrate itself functions as the “sensing layer”. While some capacitive humidity sensors depend on an additional sensing layer deposited on top of the IDE [13,23,24,31], using only a single material simplifies sensor fabrication, function, and reduces costs. This work is the first to extensively characterize PET, PLA, and potato starch as a sensing layer.

In order for a humidity sensor to be widely applicable, it must have high sensitivity over a range of humidity, quick response times, little hysteresis, temperature independence, a simple structure, and low costs to manufacture [1]. Thus, each sensor type was exposed to programmable environmental conditions in order to analyze these characteristics. Specifically, the sensor sensitivity, step response time, temperature dependency, and hysteresis are examined.

## 2. Materials and Methods

Four types of materials were used as a substrate for the sensors: 50 μm thick planarized PET from Kimoto Tech, 300 μm thick PLA from Tamiya, 300 μm thick glossy coated paper from Kelly Paper, and 500 μm thick starch and PP from Biodegradable Food Service. Prior to printing, the starch substrate was sanded to improve surface uniformity.

**Figure 1:**
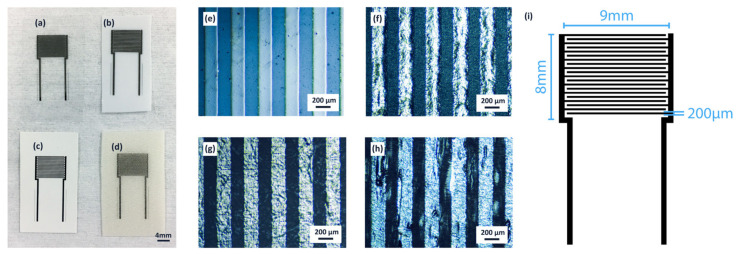
IDE capacitive sensor design and microscopic images of the respective fingers, inkjet printed in silver on (**a**,**e**) PET, (**b**,**f**) PLA, (**c**,**g**) glossy photo paper, and (**d**,**h**) starch and PP composite. (**i**) shows the print design, annotated with respective dimensions.

Figure 1i depicts the design for the interdigitated electrode sensor. Each finger is 200 μm wide with a 200 μm gap between neighboring fingers. There are 10 pairs of fingers, with a 9 mm overlap between fingers.

Sensor electrodes were printed with a drop spacing of 33 μm onto the substrates using the Dimatix Materials Printer DMP-2850 (Fujifilm, Santa Clara, CA, USA) in silver DGP 40LT-15C ink from ANP (Seoul, Korea). The platen was heated to 60 °C for printing on all four substrates to reduce ink spreading (Figure 1). The PET- and paper-based sensors were cured at 120 °C for 30 min, while the PLA- and starch-based sensors were taped to a glass slide and cured at 100 °C for 1 h to prevent warping. Minimal spreading of about 5–10 μm was observed for all four sensors.

In addition to the sensors, 1 × 1 cm^2^ squares of ink were also printed onto each substrate. The squares were cured under the same conditions as the sensors, and their conductivity was measured using a Signatone 4 point probe.

Printed sensors were trimmed to size and clipped into a flexible flat cable connector and connected to a precision LCR meter (E4980A, Agilent, Santa Clara, CA, USA) to take capacitance readings of the sensor. Capacitance measurements were taken every 12 s at 5 kHz frequency. The printed sensor, a commercially available humidity sensor sensor (RH520B, ExTech, Nashua, NH, USA), and the associated 1 × 1 cm^2^ square were placed inside of an environmental chamber (Associated Environmental Systems BHS-503, Acton, MA, USA) and subjected to a series of humidity and temperature tests. The experimental setup can be seen in the Supplementary Information, Figure S1. The entire test cycle was repeated for each sensor individually. After each exposure to humidity testing, the conductivity of the 1 × 1 cm^2^ square was measured.

**Figure 2:**
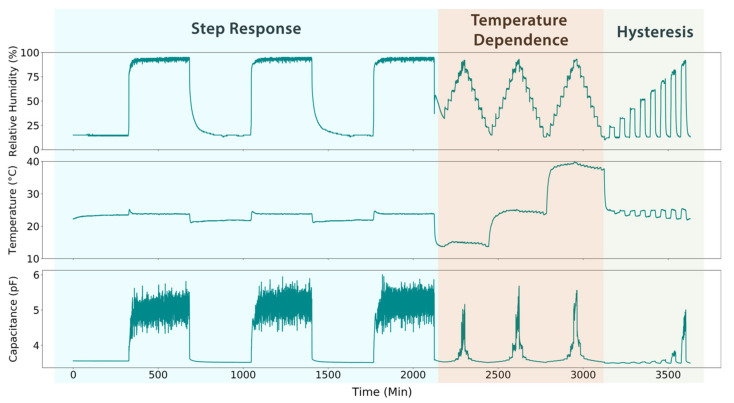
The top two plots show the relative humidity and temperature in the environmental chamber over the course of testing. The bottom plot shows the corresponding capacitive data generated by the PET-based sensor. Sensor testing is broken into three parts: step response, temperature dependence, and hysteresis.

Sensors underwent three types of tests, as plotted in Figure 2. To examine sensor step response, the capacitance of the sensors was measured while the relative humidity was raised from 15% to 95% and held constant. After 6 h, the relative humidity was reduced back to 15%, and held constant for another 6 h. This was repeated for three cycles. The effect of temperature on the sensors was examined by running identical humidity ‘pyramids’ at temperatures of 15, 25, and 40 °C. During this measurement, relative humidity increases by 10% increments, which are held for 30 min. Once 90% is reached, relative humidity is decreased by the same incremental steps. Finally, sensor hysteresis was tested by progressively ramping up relative humidity from 20% to 90% in steps of 10%. Each step was held for 30 min, and relative humidity would return to a baseline of 15% for 30 min in between steps.

Data are presented and analyzed for a single sensor on each material; however, additional data are available in the Supplementary Materials.

## 3. Results and Discussion

### 3.1. Sensitivity

**Figure 3:**
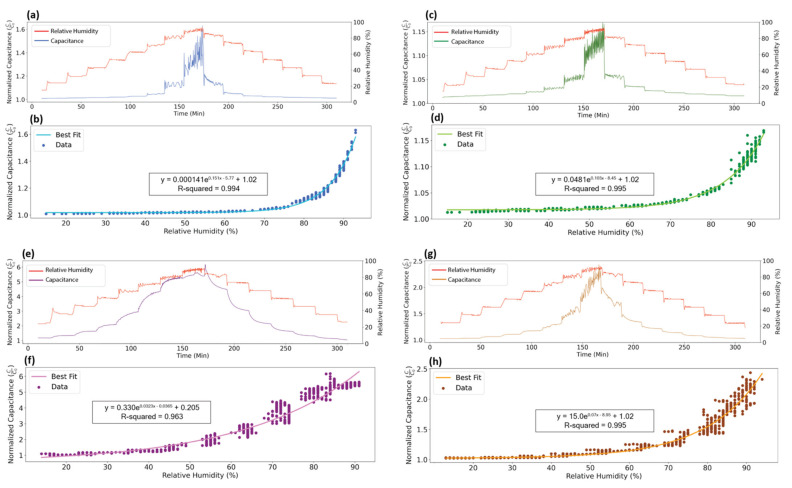
Plots of PET- (**a**,**b**), PLA- (**c**,**d**), paper- (**e**,**f**), and starch-based (**g**,**h**) sensor response. Plots (**a**,**c**,**e**,**g**) show the environmental relative humidity and the capacitive response with respect to time. Plots (**b**,**d**,**f**,**h**) take the same relative humidity data and plot them versus the corresponding capacitive response. These plots also include an optimized best fit line to describe the relationship between relative humidity and sensor capacitance.

Sensor data can be represented along a temporal x-axis or can be plotted to show the relationship between relative humidity and capacitance. Figure 3 demonstrates equivalent data plotted in these two different ways. In Figure 3a, relative humidity measured from the commercial reference and normalized capacitance data from the PET sensor is shown in relation to when it was collected in time. This type of plot allows for better visualization of sensor response to changing or constant environmental conditions. In Figure 3b, relative humidity versus normalized capacitance data from the PET sensor are plotted, with each data point corresponding to a moment in time, and the x- and y-coordinate corresponding to the respective measured humidity and capacitance at that time-point. Capacitance is normalized by dividing by the base capacitance of each sensor, which is the capacitance after 6 h exposure to 15% relative humidity and 25 °C. Normalization allows for direct comparison between different sensors and easier interpretation of capacitive output range.

Figure 3 illustrates the differences in sensitivity between the four sensors. Sensitivity is defined as the ratio of sensor output to input or change in capacitance over change in relative humidity. In Figure 3b,d,f,h, sensitivity is the slope of the line relating input humidity to output capacitance. The sensors demonstrate a similar exponential growth in sensitivity, with sensitivity greatly increasing at relative humidity above approximately 75%. In the case of the paper sensor, this threshold is closer to 60% relative humidity. Because sensitivity is highest at high humidities, the fabricated sensors capture and amplify small fluctuations in actual humidity inside the environmental chamber during the 80% and 90% RH steps.

The relationship between relative humidity and capacitance is typically non-linear for capacitive humidity sensors [17,32,33], as this relationship is dictated by the way that water vapor interacts with the substrate. Here, we propose that the relationship is captured by an exponential growth curve. Only the model for the paper sensor has a coefficient of determination below 0.99. Paper is non-polar and thus does not form polar interactions with the absorbed water molecules. However, in the polar materials, absorption increases as a function of material saturation, as interactions with the water molecules cause swelling.

Total sensitivity can be compared between sensors by looking at capacitive output range at a certain temperature. The paper- and starch-based sensors have the highest sensitivity, with capacitance increasing by a factor of 6 and 2.5, respectively, as relative humidity goes from a low of 15% to a high of 90%. High sensitivity gives paper and starch an advantage over the lower sensitivity PET- and PLA-based sensors when monitoring small changes in relative humidity. In comparison, PET and PLA only show an increase in capacitance by a factor of 1.6 and 1.15, respectively.

### 3.2. Step Response

**Figure 4:**
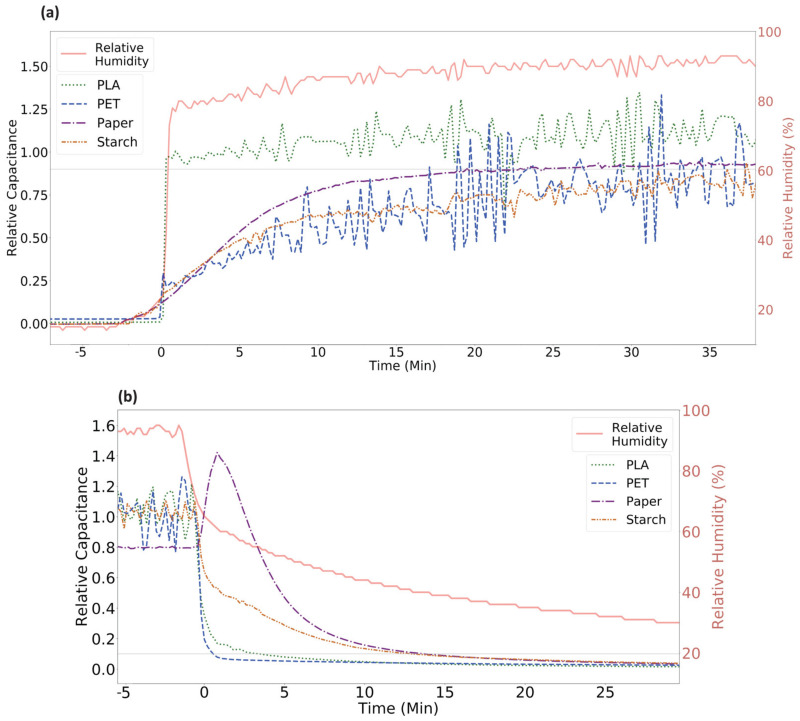
Overlaid comparison of (**a**) the response and (**b**) recovery curves for all four sensors, where the red curve corresponds to the ground-truth relative humidity in the environmental chamber. Relative capacitance is calculated using the average minimum and maximum capacitance response for each sensor.

Figure 4 shows the overlaid step response and recovery of the four humidity sensors, while Table 1 summarizes the response and recovery times. In this setup, relative humidity was increased to 90% relative humidity and then decreased to 15% relative humidity in a stepwise fashion. Relative capacitance along the y-axis is calculated using Equation (1) so that different sensors can be directly compared.

(1) Relative Capacitance=C−C¯minC¯max−C¯min

${\bar{C}}_{min}$ is the average capacitance found during the low humidity phase after the capacitance value has stabilized. ${\bar{C}}_{max}$ is the same, but calculated during the high humidity phase.

Response time is defined as the time it takes for the sensor capacitance to increase from a local minimum to within 90% of the average maximum capacitance across the high relative humidity region. Similarly, recovery time is defined as the time it takes for the sensor capacitance to decrease from a local maximum to within 10% of the average minimum capacitance across the low relative humidity region [34]. However, because the environmental chamber takes a finite, non-zero amount of time to change humidity, the humidity step is not a pure step function. This introduces a systematic error that results in longer measured response and recovery times.

Response and recovery time depends on three physical processes of permeation. When humidity increases, water vapor absorbs on the surface of material, dissolves through the surface, and diffuses throughout the material. Dissolution depends on a variety of factors such as the interaction energy between the material and water vapor, the concentration of environmental water vapor, and the saturation of the material. Diffusion is driven by a concentration gradient within the material and depends on factors including material porosity, free volume, and substrate thickness. Desorption of water vapor from the substrate occurs when the environmental humidity is low enough that it cannot maintain saturation of the material. The rate of desorption is a function of relative humidity and is not simply the reverse of absorption [18,19]. Therefore, response and recovery times tend to be different.

The PLA-based sensor has a near instant response to the humidity step, with a response time less than 1 min. A fast response time can be an important characteristic of humidity sensors when sudden environmental changes need to be detected. Meanwhile, the PET-, paper-, and starch-based sensors show a gradual response resembling a logrithmic growth curve, and response times of 21, 24, and 38 min, respectively. Both the paper and PET substrate have a protective film on their surface, which likely slows the rate at which water vapor can dissolve into the bulk of the material, and consequently increases the response time. The starch substrate has a very slow response time because it is both thicker and more porous than the others. Both characteristics allow it to absorb more water than the other substrates, which is reflected in starch’s high sensitivity, but this process takes time.

It is expected that the polar materials will have a longer recovery time than response time because of interactions between the material and the absorbed water molecules. This is only true in the case of the PLA-based sensor, which has a recovery time of 3 min. Meanwhile, the recovery time for the PET-, paper-, and starch-based sensors was shorter than their response time at 1, 13, and 13 min, respectively. Although PET is a polar material, it is possible that the planarized coating interferes with absorption, leading to a slow response time relative to recovery time. Paper is not polar, thus a slower recovery time is not expected. Another interesting feature of the paper sensor is the large spike in capacitance right as humidity is dropped from high to low. This may be due to condensation on the surface of the substrate.

### 3.3. Hysteresis

Hysteresis occurs when the capacitive output depends on whether relative humidity is increasing or decreasing to the point of measurement. The repeatability and accuracy of a sensor’s measurement depend largely on the amount of hysteresis, thus hysteresis should be minimized.

If the relative humidity around a sensor drops, but the sensor is slow to release absorbed water, the capacitance of the sensor will remain high until enough time has passed for water to desorb completely [17]. This can result in hysteresis curves, in which the sensor’s capacitance going from low to high relative humidity is smaller than when going from high to low relative humidity. The amount of sensor hysteresis depends on the humidity range as well as the rate at which humidity is changing, and thus is closely tied to the response and recovery time of the sensor.

Hysteresis was tested by cycling between a constant low (15%) and progressively increasing relative humidity. Varying the relative humidity can reveal the extent to which sensors are affected by previous humidity exposures. Hysteresis percentage was calculated for the 30%, 60%, and 90% steps using Equation (2) [35,36], where *C_i_* corresponds to capacitance during the *i*th humidity step, and the maximum and minimum capacitance values are found for a single step. The numerator is the largest capacitive difference between the curves generated by the humidity increase and decrease. The denominator captures the range of capacitance values, or the full scale deflection, in order to normalize the hysteresis percentage. Normalization is necessary when comparing between humidity steps because hysteresis is dependent on the maximum humidity reached during the step.

(2) Hysteresis= maxCi,increasing−Ci,decreasingCi,max−Ci,min

**Figure 5:**
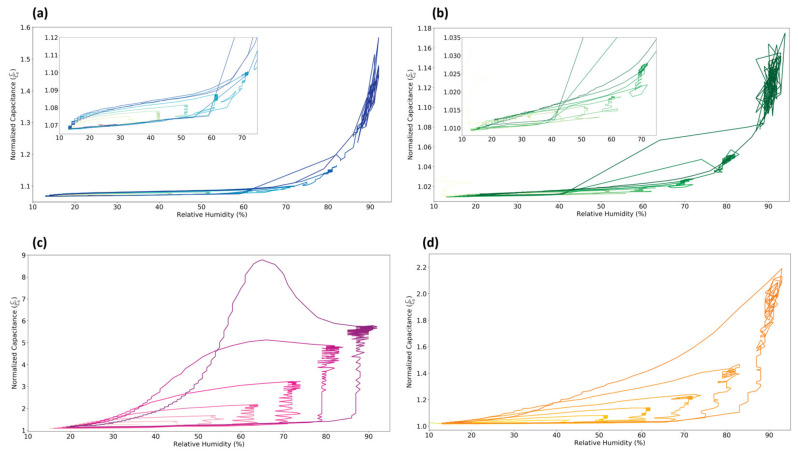
Hysteresis curves for PET- (**a**), PLA- (**b**), paper- (**c**), and starch-based (**d**) sensors. The change in color corresponds to a change in time, with colors getting darker as the test progresses.

Table 2 shows the hysteresis percentage for all sensors across three humidity steps, and Figure 5 depicts the corresponding hysteresis curves for the entire humidity step test. The relative size of each loop on the humidity versus capacitance plots approximates the amount of hysteresis occurring, with the ideal being a loop with negligible area.

The PET- and PLA-based sensors show the least amount of hysteresis, with relative hysteresis decreasing as the humidity steps increase. Small hysteresis curves can be distinguished when zooming in on the left side of the plot, but the amount of hysteresis is low compared with the capacitance range and sensitivity. Meanwhile, the paper- and starch-based sensors show considerable humidity-independent hysteresis as a result of their long response and recovery time. The difference between response and recovery rates results in asymmetric hysteresis loops, where the high capacitance recovery curve has a more gradual slope than the low capacitance response curve. Because response times are longer, most of the capacitance increase occurs in the vertical region of the plot where relative humidity is held constant. The spike in capacitance of the paper sensor as relative humidity goes from 95% to 15% is noticeable in Figure 5, while the rest of the hysteresis loops have scaled versions of the same profile. Aside from the capacitive spike, the paper- and starch-based substrates have hysteresis loops that resemble each other, likely owing to their similar response and recovery times.

### 3.4. Temperature Sensativity

To test temperature dependency, sensors were exposed to relative humidities between 15% and 95% over three distinct temperatures (15 °C, 25 °C, and 40 °C). Temperature impacts the permeation properties of the sensing layer, which can explain a sensor’s temperature dependency. Specifically, a rise in temperature corresponds to an increase in the material’s diffusion coefficient (rate of molecular diffusion) and maximum saturation concentration. This is because higher temperature leads to thermal expansion and to an increased mobility of the water molecules.

**Figure 6:**
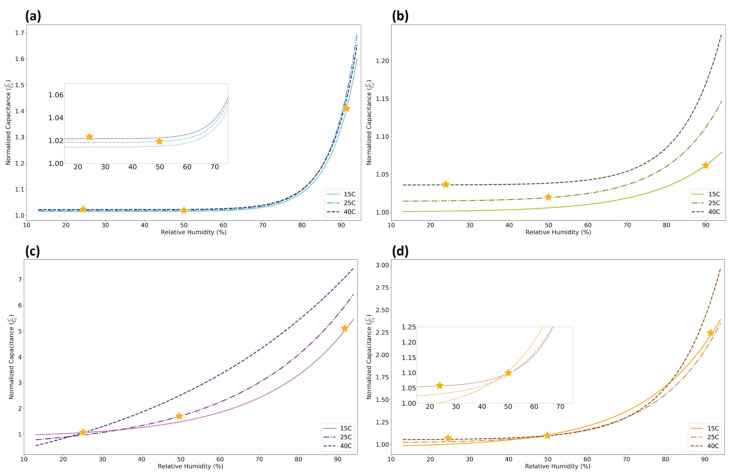
Model of relative humidity versus capacitance for PET (**a**), PLA (**b**), paper (**c**), and starch (**d**) substrates during humidity cycles performed at three different temperatures. Models were generated based on real data. Star markers on each curve represent relative humidity and temperature combinations that result in the same absolute humidity of 0.015 kg/m^3^.

Figure 6 uses the exponential growth models described in the sensitivity section in order to model the relationship with reduced noise. If a sensor is highly affected by temperature, then the three temperature curves will have a significant spread. A higher temperature should lead to higher capacitive outputs, which is mainly true for all four sensors. The change in capacitance as a function of temperature is called “thermal drift”, and is given by Equation (3) [37]. Table 3 summarizes the thermal drift calculated between the 15 °C, 25 °C, and 40 °C curves for sensors at 30%, 60%, and 90% relative humidity. To account for noise in the data, the capacitance used was an average value taken over the range of ±3% of the relative humidity of interest. Table S1 in the Supplementary Materials shows the thermal drift calculated using the models rather than the raw data.

(3) Thermal Drift=∆C∆T

All four sensors show an increase in thermal drift as relative humidity increases. This suggests that sensitivity at high relative humidity is significantly temperature-dependent, which can be explained by the changes in material permeation as a function of temperature. PET and starch appear to be the least affected by temperature changes, with little variance in sensitivity and base capacitance throughout the three temperature cycles. Paper is affected by temperature, with slight increases in curve slope corresponding to increased temperatures. Although thermal drift is relatively high for this material, paper has a large operating range compared with the polymer materials. Meanwhile, the effects of temperature on the permeation properties and thus sensitivity of the sensors are most evident for the PLA-based sensor, where the curve slope and maximum capacitance increase significantly with temperature.

Examining sensor temperature dependence is necessary to establish if sensors are measuring relative humidity or something else. A common misconception is that these humidity sensors only measure the amount of water vapor in the air, or the absolute humidity. At standard pressure, the capacitance output for 15 °C air at 90% relative humidity, 25 °C air at 50% relative humidity, and 40 °C air at 24% relative humidity should be the same regardless of the temperature difference, as these conditions have the same absolute humidity. However, the star markers in Figure 6 show that these conditions result in different capacitance outputs for each sensor. These sensors are temperature dependent, and thus are measuring relative humidity rather than absolute humidity.

### 3.5. Material Conductivity

In order to determine whether short-term humidity exposure degrades the integrity of the sensors, the 1 × 1 cm^2^ printed test square was put in the humidity chamber alongside the corresponding sensor, and its conductivity was measured after each test cycle using a 10 μm source current. Table 4 shows the resistivity of the squares before and after exposure to humidity testing. Although sintered at a lower temperature for a longer period of time, the printed square on the PLA substrate has a similar resistivity to that of the PET and paper samples. The starch sample was cured at the same temperature and for the same amount of time as the PLA sample, but its resistivity is significantly higher. This is likely owing to surface roughness rather than different curing conditions. After undergoing two humidity tests, a total of 120 h, the resistivity of the test squares remained fairly constant. These data support that certain biodegradable humidity sensors can support short-term use without a large decrease in conductivity.

## 4. Conclusions

Recent innovations in smart packaging for manufacturer to consumer tracking, as well as high density monitoring of crop conditions, require the development of biodegradable humidity sensors. A widely applicable sensor has high sensitivity, fast response times, little hysteresis, and temperature independence. Each of the four studied sensors excel in some, but not all of these categories, and thus would work best for specific rather than general applications. Table 5 shows a comprehensive comparison of all these properties. The PET-based sensors prove to be temperature independent with repeatable outputs that are not significantly affected by hysteresis. However, this sensor has low sensitivity at lower relative humidity, and is not biodegradable. It could work best in environments where a range of temperatures and only high humidity is expected. Overall, the PLA-based sensors appear the most versatile, with fast response times, limited hysteresis, and biodegradability. PLA is a popular material for packaging, and humidity sensors could be integrated directly into the packaging to monitor the conditions of the contents. Paper-based sensors are limited because of long response times; however, in environments where humidity changes gradually, these sensors are quite viable. Additionally, paper is an appealing substrate because of its versatility; it can be folded, cut, and taped into any form, and it can be recycled upon use and is also a common packaging material. Finally, starch-based substrates are similarly limited by slow response times and hysteresis, but also demonstrate high sensitivity and temperature stability. This substrate shows the potential for future plant-based sensors as renewable materials become more widespread.

Although this paper supports the viability of biodegradable humidity sensors for short-term use and establishes a baseline performance for newly fabricated devices, future work could look at the change in performance over long-scale testing. It can be expected that sensing behavior will change as the substrate begins to break down. The method of degradation, either through hydrolysis, bacterial activity, or other methods, might also impact long-term sensor behavior. In addition to extended tracking of sensor performance, future tests could include examining a wider range of temperature stability, using fully biocompatible inks, and integrating the sensor with transducers for easier data collection.

## Figures and Tables

**Table 1:** Comparison of the response and recovery times for each sensor.

Material	Response Time (min)	Recovery Time (min)
PET	17	1
PLA	<1	3
Paper	21	13
Starch	38	13

**Table 2:** Comparison of the percent hysteresis for each sensor at three different relative humidity steps.

Material	30% RH Hysteresis (%)	60% RH Hysteresis (%)	90% RH Hysteresis (%)
PET	67.5	53.4	9.20
PLA	54.6	55.8	34.3
Paper	101	109	178
Starch	99	84	104

**Table 3:** Comparison of thermal drift for each sensor at three different relative humidity values. Calculated from raw data.

Materials	ΔTemperature	30% RH Thermal Drift (fF/°C)	60% RH Thermal Drift (fF/°C)	90% RH Thermal Drift (fF/°C)
PET				
	25 °C–15 °C	1.17	1.73	14.5
	40 °C–25 °C	1.27	1.23	−4.93
	40 °C–15 °C	1.22	1.52	4.41
PLA				
	25 °C–15 °C	7.14	8.56	25.2
	40 °C–25 °C	5.82	6.76	17.6
	40 °C–15 °C	6.36	7.36	20.7
Paper				
	25 °C–15 °C	−19.9	132	306
	40 °C–25 °C	−0.69	304	67.2
	40 °C–15 °C	−7.68	214	118
Starch				
	25 °C–15 °C	2.93	−3.06	−2.39
	40 °C–25 °C	0.488	3.88	30.7
	40 °C–15 °C	1.34	0.918	9.34

**Table 4:** Resistivity measurements of 1 × 1 cm^2^ square test prints after repeated exposure to humidity cycling.

Material	Resistivity, 0 Cycles (Ω/Square)	Resistivity, 1 Cycle (Ω/Square)	Resistivity, 2 Cycles (Ω/Square)
PET	2.62	2.71	1.86
PLA	2.40	2.00	1.81
Paper	7.61	12.6	11.2
Starch	303	237	255

**Table 5:** Comparison of sensor properties.

Property	PET	PLA	Starch	Paper
Biodegradable	X	✓	✓	✓
High Sensitivity	X	X	✓	✓
Fast Response and Low Hysteresis	✓	✓	X	X
Low Thermal Drift	✓	X	✓	X

## Data Availability

The data presented in this study are available on request from the corresponding author.

## Supplementary Materials

The following are available online at https://www.mdpi.com/article/10.3390/s21196557/s1, Figure S1: Experimental setup. AES Environmental Chamber is used to control relative humidity and temperature conditions. Reference data is collected via the commercially available Extech Humidity Meter. IDE capacitance of the fabricated sensor is measured using an LCR Meter. Finally, humidity, temperature, and capacitance data is sent to a computer for analysis, Figure S2: Additional device data. Each row shows data from a different device, and each column highlights a specific characteristic. Table S1: Comparison of thermal drift for each sensor at three different relative humidity values. Calculated from models.

## References

[1] Yamazoe N., Shimizu Y. (1986). Humidity sensors: Principles and applications. Sens. Actuators.

[2] Nathan A., Ahnood A., Cole M.T., Lee S., Suzuki Y., Hiralal P., Bonaccorso F., Hasan T., Garcia-Gancedo L., Dyadyusha A. (2012). Flexible electronics: The next ubiquitous platform. Proc. IEEE.

[3] Gaspar C., Olkkonen J., Passoja S., Smolander M. (2017). Paper as active layer in inkjet-printed capacitive humidity sensors. Sensors.

[4] Kojić T., Stojanović G.M., Miletić A., Radovanović M., Al-Salami H., Arduini F. (2019). Testing and characterization of different papers as substrate material for printed electronics and application in humidity sensor. Sens. Mater..

[5] Khan M.U., Saqib Q.M., Hassan G., Bae J. (2020). All printed organic humidity sensor based on egg albumin. Sens. Bio-Sens. Res..

[6] Sajid M., Aziz S., Kim G.B., Kim S.W., Jo J., Choi K.H. (2016). Bio-compatible organic humidity sensor transferred to arbitrary surfaces fabricated using single-cell-thick onion membrane as both the substrate and sensing layer. Sci. Rep..

[7] Müller P., Schmid M. (2019). Intelligent packaging in the food sector: A brief overview. Foods.

[8] Sani M.A., Azizi-Lalabadi M., Tavassoli M., Mohammadi K., McClements D.J. (2021). Recent advances in the development of smart and active biodegradable packaging materials. Nanomaterials.

[9] Rodrigues C., Souza V.G.L., Coelhoso I., Fernando A.L. (2021). Bio-based sensors for smart food packaging—Current applications and future trends. Sensors.

[10] Schaefer D., Cheung W.M. (2018). Smart Packaging: Opportunities and Challenges. Procedia CIRP.

[11] Dahal S., Yilma W., Sui Y., Atreya M.B., Bryan S., Davis V., Whiting G.L., Khosla R. (2020). Degradability of biodegradable soil moisture sensor components and their effect on maize (*Zea mays* L.) growth. Sensors.

[12] Sui Y., Atreya M., Dahal S., Gopalakrishnan A., Khosla R., Whiting G.L. (2021). Controlled Biodegradation of an Additively Fabricated Capacitive Soil Moisture Sensor. ACS Sustain. Chem. Eng..

[13] Ding H., He P., Yang J., Liu C., Zhao H., Derby B. (2020). Water-based highly conductive graphene inks for fully printed humidity sensors. J. Phys. D Appl. Phys..

[14] Fernandez F.D.M., Bissannagari M., Kim J. (2021). Fully inkjet-printed BaTiO3 capacitive humidity sensor: Microstructural engineering of the humidity sensing layer using bimodal ink. Ceram. Int..

[15] Farahani H., Wagiran R., Hamidon M.N. (2014). Humidity sensors principle, mechanism, and fabrication technologies: A comprehensive review. Sensors.

[16] Igreja R., Dias C.J. (2004). Analytical evaluation of the interdigital electrodes capacitance for a multi-layered structure. Sens. Actuators A Phys..

[17] Leondes C. (2006). MEMS/NEMS Handbook Techniques and Applications.

[18] Duncan B., Urquhart J., Roberts S. (2005). Review of Measurement and Modelling of Permeation and Diffusion in Polymers.

[19] Duncan B.C., Broughton W.R. (2007). Absorption and Diffusion of Moisture in Polymeric Materials.

[20] Chen Z., Lu C. (2005). Humidity sensors: A review of materials and mechanisms. Sens. Lett..

[21] Engarnevis A., Romani S., Sylvester A., Huizing R., Green S., Rogak S. The Effects of Temperature and Humidity on the Permeation Properties of Membrane Transport Media Used in Energy Recovery Ventilators. Proceedings of the ASHRAE 2017 Annual Conference.

[22] Jansen K.M.B., Zhang M.F., Ernst L.J., Vu D.K., Weiss L. (2020). Effect of temperature and humidity on moisture diffusion in an epoxy moulding compound material. Microelectron. Reliab..

[23] Sulaiman S., Rashid N.A., Aziz A.S.A., Jun L.Q., Jaafar S.M.H.S.M. Inkjet-Printed Graphene-Based Flexible Humidity Sensor for Environmental Applications. Proceedings of the 2020 IEEE International Conference on Semiconductor Electronics (ICSE).

[24] Molina-Lopez F., Briand D., De Rooij N.F. (2012). All additive inkjet printed humidity sensors on plastic substrate. Sens. Actuators B Chem..

[25] Zaaba N.F., Jaafar M. (2020). A review on degradation mechanisms of polylactic acid: Hydrolytic, photodegradative, microbial, and enzymatic degradation. Polym. Eng. Sci..

[26] Teixeira S., Eblagon K.M., Miranda F., Pereira M.F.R., Figueiredo J.L. (2021). Towards Controlled Degradation of Poly(lactic) Acid in Technical Applications. C.

[27] Casalini T., Rossi F., Castrovinci A., Perale G. (2019). A Perspective on Polylactic Acid-Based Polymers Use for Nanoparticles Synthesis and Applications. Front. Bioeng. Biotechnol..

[28] Kumar S., Bhushan P., Agarwal A.K., Bhattacharya S. (2019). Paper Based Sensors for Environmental Monitoring. A Historical Perspective on Paper Microfluidic Based Point-of-Care Diagnostics.

[29] Shahani C.J., Harrison G. (2002). Spontaneous Formation of Acids in the Natural Aging of Paper. Stud. Conserv..

[30] Jiang T., Duan Q., Zhu J., Liu H., Yu L. (2020). Starch-based biodegradable materials: Challenges and opportunities. Adv. Ind. Eng. Polym. Res..

[31] Zhou R., Li J., Jiang H., Li H., Wang Y., Briand D., Camara M., Zhou G., de Rooij N.F. (2019). Highly transparent humidity sensor with thin cellulose acetate butyrate and hydrophobic AF1600X vapor permeating layers fabricated by screen printing. Sens. Actuators B Chem..

[32] Kim Y., Jung B., Lee H., Kim H., Lee K., Park H. (2009). Capacitive humidity sensor design based on anodic aluminum oxide. Sens. Actuators B Chem..

[33] Lee C.Y., Lee G. (2005). Bin Humidity sensors: A review. Sens. Lett..

[34] Koncar V. (2019). Structural Health Monitoring of Processes Related to Composite Manufacturing.

[35] Bolton W. (2021). Instrumentation and Control Systems.

[36] Purdue University (2011). Static Calibration. Measurement Systems.

[37] Romero F.J., Rivadeneyra A., Salinas-Castillo A., Ohata A., Morales D.P., Becherer M., Rodriguez N. (2019). Design, fabrication and characterization of capacitive humidity sensors based on emerging flexible technologies. Sens. Actuators B Chem..

