# Supplementary material for: Characterization and Comparison of Biodegradable Printed Capacitive Humidity Sensors
Source: Sensors (Basel). 2021 Sep 30;21(19):6557. doi: 10.3390/s21196557 (PMC8512811; doi:10.3390/s21196557)
Supplement: Supplementary file 1 [file sensors-21-06557-s001.zip › sensors-1366467-supplementary.pdf]

# Supplementary Material

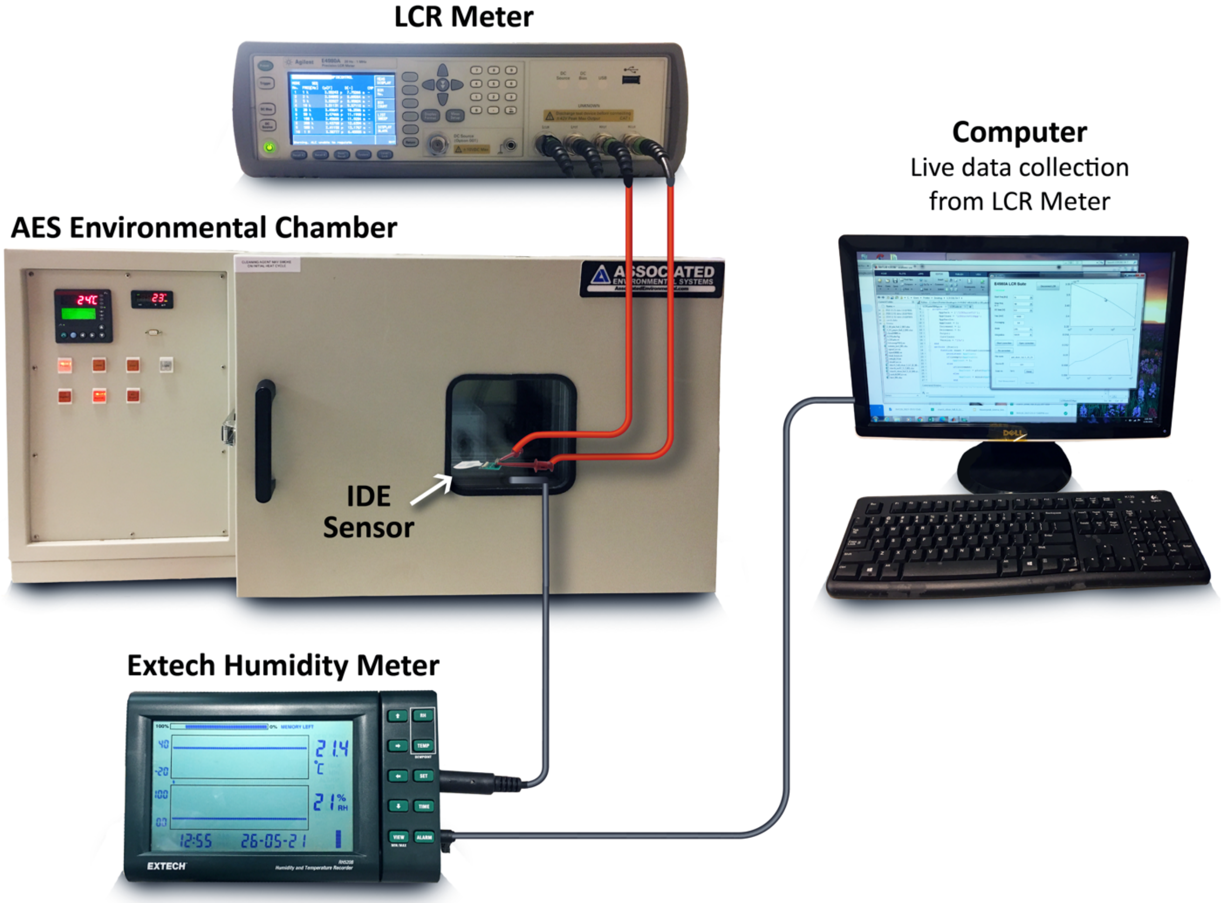


**Figure S1.** Experimental setup. AES Environmental Chamber is used to control relative humidity and temperature conditions. Reference data is collected via the commercially available Extech Humidity Meter. IDE capacitance of the fabricated sensor is measured using an LCR Meter. Finally, humidity, temperature, and capacitance data is sent to a computer for analysis.

**Table S1.** Comparison of thermal drift for each sensor at three different relative humidity values. Calculated from models.

| Materials | ΔTemperature | 30%RH Thermal Drift (fF/°C) | 60%RH Thermal Drift (fF/°C) | 90%RH Thermal Drift (fF/°C) |
|---|---|---|---|---|
| **PET** | | | | |
| | 25°C - 15°C | 1.46 | 1.42 | 16.3 |
| | 40°C - 25°C | 1.10 | 1.19 | -5.49 |
| | 40°C - 15°C | 2.57 | 2.56 | 10.8 |
| **PLA** | | | | |
| | 25°C - 15°C | 6.41 | 6.84 | 23.7 |
| | 40°C - 25°C | 9.86 | 8.46 | 27.3 |
| | 40°C - 15°C | 16.3 | 15.3 | 51.1 |
| **Paper** | | | | |
| | 25°C - 15°C | -19.0 | 165 | 388 |
| | 40°C - 25°C | 94.8 | 446 | 487 |
| | 40°C - 15°C | 75.8 | 611 | 875 |
| **Starch** | | | | |
| | 25°C - 15°C | 2.89 | -4.92 | -9.21 |
| | 40°C - 25°C | 2.84 | -0.876 | 46.0 |
| | 40°C - 15°C | 5.73 | -5.801 | 36.8 |

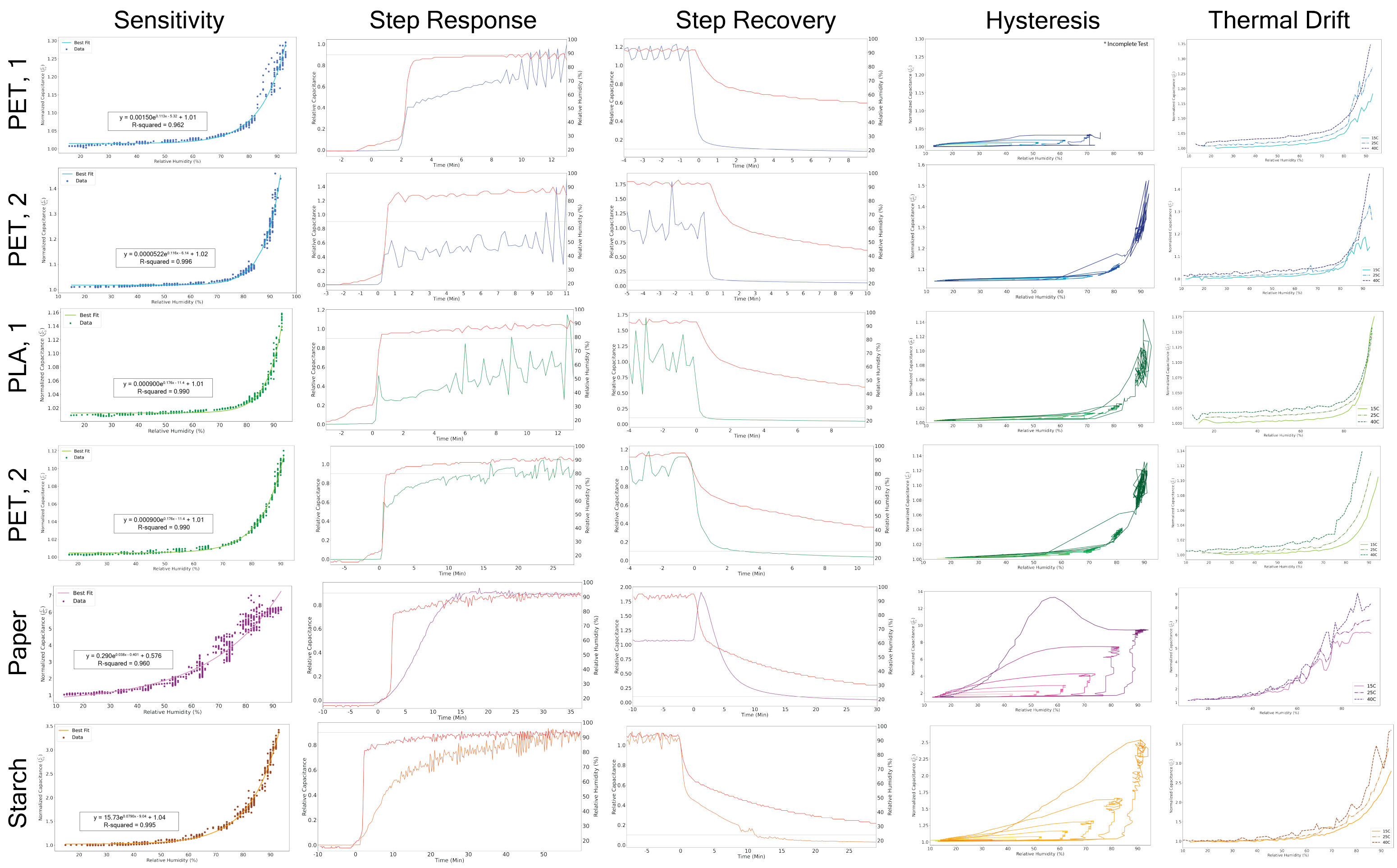


**Figure S2.** Additional device data. Each row shows data from a different device, and each column highlights a specific characteristic.
